# The Diagnosis of Congestive Heart Failure Based on Generalized Multiscale Entropy-Wavelet Leaders

**DOI:** 10.3390/e24121763

**Published:** 2022-12-01

**Authors:** Juanjuan Yang, Caiping Xi

**Affiliations:** 1 Ocean College, Jiangsu University of Science and Technology, Zhenjiang 212100, China; 2 College of Automation, Jiangsu University of Science and Technology, Zhenjiang 212100, China

**Keywords:** electrocardiogram, the generalized multiscale entropy, wavelet leaders, congestive heart failure, extreme learning machine

## Abstract

Congestive heart failure (CHF) is a chronic heart condition associated with debilitating symptoms that can lead to mortality. The electrocardiogram (ECG) is a noninvasive and simple diagnostic method that can show detectable changes in CHF. However, manual diagnosis of ECG signals is often erroneous due to the small amplitude and duration of the ECG signals. This paper presents a CHF diagnosis method based on generalized multiscale entropy (MSE)-wavelet leaders (WL) and extreme learning machine (ELM). Firstly, ECG signals from normal sinus rhythm (NSR) and congestive heart failure (CHF) patients are pre-processed. Then, parameters such as segmentation time and scale factor are chosen, and the multifractal spectrum features and number of ELM hidden layer nodes are determined. Two different data sets (A, B) were used for training and testing. In both sets, the balanced data set (B) had the highest accuracy of 99.72%, precision, sensitivity, specificity, and F1 score of 99.46%, 100%, 99.44%, and 99.73%, respectively. The unbalanced data set (A) attained an accuracy of 99.56%, precision of 99.44%, sensitivity of 99.81%, specificity of 99.17%, and F1 score of 99.62%. Finally, increasing the number of ECG segments and different algorithms validated the probability of detection of the unbalanced data set. The results indicate that our proposed method requires a lower number of ECG segments and does not require the detection of R waves. Moreover, the method can improve the probability of detection of unbalanced data sets and provide diagnostic assistance to cardiologists by providing a more objective and faster interpretation of ECG signals.

## 1. Introduction

Heart failure is a serious global public health problem caused by structural or physical dysfunction. It is also considered to be the final stage in the development of most cardiovascular diseases. Over 26 million people worldwide currently suffer from heart failure, and 70% of CHF cases are caused by cardiovascular diseases, such as coronary artery disease [1]. Other causes of CHF include an elevated hemodynamic load, dysfunction related to ischemia, adverse ventricular remodeling, and genetic mutations [2]. Notably, the prevalence of heart failure increases significantly with increasing age. Therefore, there is a need for early detection of CHF in the aging population, a problem currently faced by many countries around the world. Early detection of CHF to avoid further structural or functional damage to the heart is essential and can save lives.

The diagnosis of CHF is a clinical diagnosis that requires a combination of signs and symptoms and conclusive evidence from investigative tests. Standard diagnostic tests for CHF include chest x-ray, magnetic resonance imaging (MRI), nuclear imaging, echocardiography, and invasive angiography, which can be time-consuming and expensive [3]. However, the electrocardiogram (ECG) is a non-invasive test that has been established as central to diagnosing cardiovascular pathology. It reflects the electrical activity of the heart, and ECG is inexpensive and widely available. The ECG of CHF may be subtly altered, but any alteration in normal is not specific to the diagnosis of CHF, and most academics currently use the ECG signals to diagnose CHF. Due to the small amplitude and duration of the ECG signals, manual diagnosis of the ECG signals is often erroneous, so quantifying ECG signals can improve the objectivity and reliability of heart failure ECG signals diagnosis.

In 2002, Costa et al. [4] improved the sample entropy by introducing the concept of multiscale entropy (MSE), which assesses the complexity of a time series by quantifying the entropy of the time series over a range of time scales. Since MSE was originally introduced, it has become a popular method for quantifying signal complexity and has been successfully applied to different research areas, such as biomedical time series. The basis and implementation of multiscale entropy were subsequently described in detail in 2005, showing its applicability to human heartbeat fluctuations under physiological and pathological conditions [5]. In 2014, Wu et al. [6] proposed a composite multiscale entropy that could solve the accuracy problem of MSE and improve the accuracy of MSE estimation. In 2015, Heurtier [7] elaborated on the MSE algorithm and an improved MSE estimation algorithm and extended MSE to higher-order moments. In the same year, Gao et al. [8] investigated the fundamental bi-scaling law of fractal time series based on MSE, distinguishing healthy individuals from patients with life-threatening congestive heart failure. Costa et al. [9] extended multiscale entropy to generalized multiscale entropy by using different moments of the distribution of random variables to coarse-grain the original time series. In 2017, Liu et al. [10] proposed an MSE analysis method for differential RR interval time series signals and distinguished normal sinus rhythm subjects from CHF patients. However, it has been suggested that entropy methods often do not perform satisfactorily when they are used to analyze the non-linear complexity in physiological signals, and the choice of coarse-grained time scales leads to inaccurate estimates of entropy.

Given the non-linear dynamism of the heart and its self-similarity features, the ECG signals present fractal properties, and the use of fractal methods can also quantify time series and investigate the characteristics of the ECG signals [11]. In 2007, Makowiec et al. [12] analyzed ECG signals during the RR interval using multifractal algorithms. They analyzed the scaling properties of average multifractal partition functions in these physiologically grounded interbeat intervals: low frequency (LF), very low frequency (VLF), and ultra-low frequency (ULV), following normal RR intervals in 39 healthy subjects. In 2016, Chakraborty [13] studied ECG signals collected from the MIT-BIH database in epileptic patients and healthy individuals using the monofractal approach and multifractal approach. The results of the multifractal detrended fluctuation analysis (MFDFA) confirmed that the degree of multifractality was higher in healthy normal individuals compared to people with epilepsy. In 2021, Rogelio et al. [14] introduced a new method based on a clever fusion of fractal dimensional (FD) algorithms and fuzzy logic systems for the automatic prediction of sudden cardiac death events with an accuracy of 91.54%. Serrano et al. [15] used Cantor Set and electroencephalogram (EEG) cases to show that estimation with wavelet leaders (WL) was more accurate than estimation with MFDFA, and the computational complexity of the WL algorithm is significantly lower than that of the MFDFA algorithm. In 2019, Jahmunah et al. [16] reviewed existing methods for automatic CHF diagnosis and suggested that the use of entropic and nonlinear features has greater benefit for the automatic diagnosis of CHF from ECG signals. Therefore, this paper proposes a CHF diagnosis method based on an extreme learning machine (ELM) by a feature extraction method using a combination of entropy and multifractal algorithm and verifies the possibility and effectiveness of the proposed algorithm on ECG signals.

The amplitude of the ECG signals is measured in millivolts, and many researchers extract the R-wave in the ECG signals before extracting the features for study. There is no doubt that R-peak detection is time-consuming. Inspired by Acharya et al. [17], they summarized the methods to automatically diagnose congestive heart failure. In order to improve the classification effect, the paper proposes a CHF diagnosis method based on generalized MSE-WL and ELM. Firstly, the ECG signal amplitude is pre-processed. Secondly, features are extracted, and suitable features are selected according to the proposed method. Next, a machine learning algorithm is used to differentiate between normal and heart failure patients. The dataset is augmented to verify the accuracy of the CHF diagnosis in the unbalanced dataset. Finally, a comparison is made with different algorithms that have been used in recent years to diagnose CHF. Compared with other algorithms, the combination of generalized MSE and WL algorithm features not only improves the diagnosis rate of CHF but also does not require the execution of R-peak detection.

## 2. Materials and Methods

### 2.1. Materials

In this study, we used data from three different databases of Physionet. These are from Beth Israel Deaconess Medical Center (BIDMC), Congestive Heart Failure Database (CHF), and MIT-BIH Normal Sinus Rhythm Database (NSR). The CHF and NSR databases are described in detail as follows:

BIDMC congestive heart failure database: The following database has more prolonged ECG recordings (NYHA Class 3 and Class 4) from 15 patients (11 men and 4 women) aged 22 to 71 with severe congestive heart failure [18]. The 20-h recording of each subject contains two ECG signals with a sampling frequency of 250 Hz. Two recordings of each subject constitute 15×2=30 recordings of the CHF database used in this study.

MIT-BIH normal sinus rhythm database: It contains 25 h of ECG recordings from 18 subjects aged 20 to 50, 5 men and 13 women, from the Arrhythmia Laboratory at Beth Israel Hospital in Boston [19]. Here, data were acquired at a sampling frequency of 128 Hz. Two ECG signals were available for each subject. Notably, subjects in this database were found to have no significant arrhythmias. Two recordings of each subject constitute 18×2=36 recordings of the NSR database used in this study.

**Figure 1:**
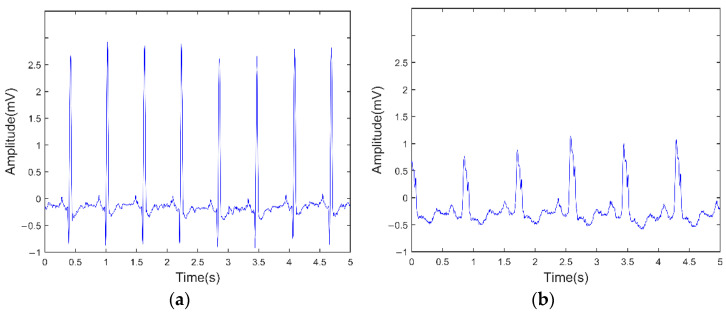
Raw ECG signals. (**a**) Normal ECG signal; (**b**) CHF ECG signal.

This paper analyses 18×2+15×2=66 recordings of NSR data and CHF data, which were obtained from ECG recordings as raw data (in mV). They are then segmented into 5 s ECG (without R-wave extraction). A Normal ECG signal and the ECG signal of a CHF patient are shown in Figure 1a,b.

### 2.2. Methods

**Figure 2:**
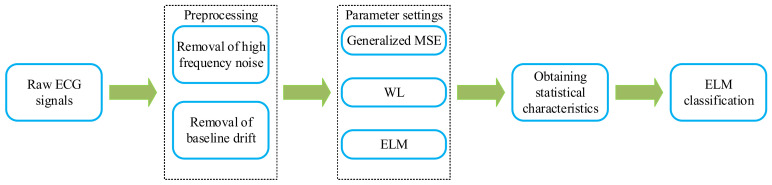
Block diagram of the ECG signals classification system proposed in this paper.

This study focuses on proposing an automatic CHF detection method based on the generalized multiscale entropy-wavelet leaders and extreme learning machine. The technique used for ECG signals classification and the steps can be divided into five parts in Figure 2. Firstly, the original ECG signals are extracted from the physiological database. Next, preprocessing is performed to remove noise and baseline drift. Then, the effect of parameter settings on the model is obtained according to the generalized MSE and the WL algorithms. Subsequently, suitable parameters are selected to obtain statistical features. Finally, the features are fed into the trained model for the classification and diagnosis of CHF patients.

#### 2.2.1. Pre-Processing

In practice, ECG signals are negatively affected by many unfavorable factors during data acquisition and transmission, leading to signal bias and inaccurate diagnosis. Many different algorithms [20,21,22] have been proposed to suppress noise and obtain clean ECG signals. The wavelet transform, with its different scales and low entropy, is highly advantageous in non-stationary signal processing [23]. In this paper, high-frequency noise and baseline drift in the original ECG signals are removed by wavelet methods.

The raw ECG signals contain high-frequency noise and baseline drift, which can be removed by wavelet methods. In this paper, we have performed an 8-level decomposition of the ECG signals. This is because the decomposition levels were chosen to be high to ensure the presence of any low-frequency components of the main signals. We use the ‘bior2.6’ wavelet basis function to obtain a reconstructed denoised signal that has no baseline drift.

**Figure 3:**
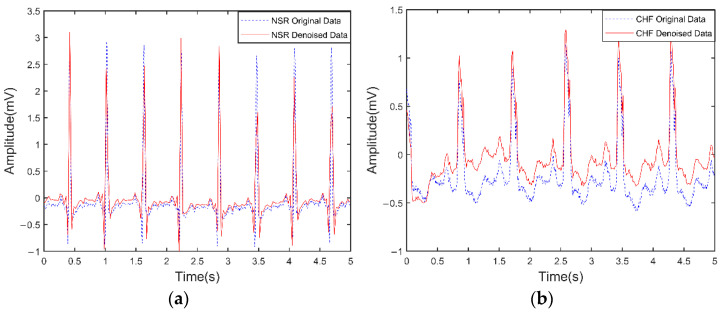
ECG signals with attenuated noise. (**a**) Normal ECG signal; (**b**) CHF ECG signal. The wavelet basis function is ‘bior2.6’ and the number of wavelet layers is 8.

The waveforms before and after the wavelet denoising of NSR and CHF are shown in Figure 3. The ECG signals are split into segments of the time length of 5 s, and it is clear from Figure 3a,b that the small fluctuations in the waveform between the large fluctuations have been removed. Since we use the ‘bior2.6’ wavelet basis function with 8-layer decomposition, the length of the time series should be greater than 256.

#### 2.2.2. Multiscale Entropy Algorithm with the First Moment

Considering a denoised time series $\left\{x_{1},x_{2},\cdots ,x_{N}\right\}$, the generalized MSE is calculated as follows: First, the original signals are divided into mutually disjoint segments of the length s. Second, selected moments are estimated for each of these segments to produce a coarse-grained time series of scale s. Finally, sample entropy is calculated for each coarse-grained time series.

The multiscale entropy algorithm with the first moment can be denoted as MSEN1; the steps are as follows [7,8]:

**Step 1.** For a time series $\left\{x_{1},x_{2},\cdots ,x_{N}\right\}$ of length N, t=1,2,⋯,N, construct a new sequence $y_{j}$ by dividing the original time series into non-overlapping segments of length s, try to use the equation $y_{j}=\frac{1}{s}\sum_{i=(j-1)*s}^{js}x_{i}$ to obtain the average of each interval of length s, we can obtain the new time series $\left\{y_{j},j=1,2,\cdots ,N/s\right\}$ with the length $N_{s}=\left\lfloor N/s\right\rfloor$, $\left\lfloor N/s\right\rfloor$ means the largest integer not greater than N/s. When s=1, the sequence is the original time signals, and the length of the coarse-graining signals is equal to the ratio of the length of original signals to the scale s.

**Step 2.** For the new times series $\left\{y_{j},j=1,2,\cdots ,N_{s}\right\}$, try to obtain the sample entropy. Construct the m-dimensional vector of sequent m points by using the moving window with length m. Divide the time series $\left\{y_{j},j=1,2,\cdots ,N_{s}(N_{s}=N/s)\right\}$ into $N_{s}-m+1$ overlapped sequences, denoted as $X_{m}(i)=[y_{i},y_{i+1},\cdots ,y_{i+m-1}]$ which represents a vector of m consecutive values of y starting from the ith point.

**Step 3.** Define $d[X_{m}(i),X_{m}(j)]$ as the maximum distance between the corresponding elements of vectors $X_{m}(i)$ and $X_{m}(j)$, $d[X_{m}(i),X_{m}(j)]=\max|y_{i+k}-y_{j+k}|$, k∈[0,m−1], $i,j\in [1,N_{s}-m+1],i\neq j$.

**Step 4.** For a given threshold value r, generally, 10–20% [24] of the standard deviation of the time series participating in the sample entropy calculation, and the standard deviation is σ=1(Ns−1)∑i=1Ns(yi−y¯)2, y¯=1(Ns−1)∑i=1Nsyi. When $i\leq N_{s}-m+1$, count the number of values $d[X_{m}(i),X_{m}(j)]<r$ (called template matches), and take the ratio of this number $N^{m}(i)$ to the total number $N_{s}-m+1$ of distances, $A_{r}^{m}(i)=N^{m}(i)/(N_{s}-m+1)$, average it over all i indexes, we can obtain $A^{m}(r)=[1/(N_{s}-m+1)]\sum_{i=1}^{N_{s}-m+1}A_{r}^{m}(i)$.

**Step 5.** Divide the new time series $\left\{y_{j},j=1,2,\cdots ,N_{s}\right\}$ by moving widow with length m+1 to obtain overlapped $N_{s}-m$ segments, denoted as $X_{m+1}(i)=[y_{i},y_{i+1},\cdots ,y_{i+m}],$ $i=[1,N_{s}-m]$, consisting of successive m+1 values of $y_{j}$ from the ith point.

Repeat **Steps 3–4** for **Steps 6–7** to obtain the average of all i as $A^{m+1}(r)=[1/(N_{s}-m)]\sum_{i=1}^{N_{s}-m}A_{r}^{m+1}(i)$.

**Step 8**. Calculate the sample entropy:

(1) $$MSEN1=S\mathrm{amp}En(m,r,s)=-\ln[A^{m+1}(r)/A^{m}(r)].$$

#### 2.2.3. Multiscale Entropy Algorithm with the Second Moment

The multiscale entropy algorithm with the second moment by using the unbiased estimator of variance can be denoted as MSEN2, the steps are as follows.

**Step 1.** For a time series $\left\{x_{1},x_{2},\cdots ,x_{N}\right\}$ of length N, t=1,2,⋯,N, construct a new sequence $y_{j}$ by dividing the original time series into non-overlapping segments of length s, try to use the equation $y_{t}=\frac{1}{s-1}\overset{ts}{\underset{j=(t-1)s+1}{\sum }}{\left\{x_{i}-\frac{1}{s}\overset{ts}{\underset{j=(t-1)s+1}{\sum }}x_{j}\right\}}^{2}$ $,t=1,2,\ldots ,N_{s}=\left\lfloor N/s\right\rfloor$ to obtain the second moment of each segment of length s by using the unbiased estimator of variance, we can obtain the new time series $\left\{y_{j},j=1,2,\cdots ,N/s\right\}$ with the length $N_{s}=\left\lfloor N/s\right\rfloor$.

**Steps 2–7** are the same as Section 2.2.2.

**Step 8.** Calculate the sample entropy:

(2) $$MSEN2=S\mathrm{amp}En(m,r,s)=-\ln[A^{m+1}(r)/A^{m}(r)].$$

#### 2.2.4. Wavelet Leaders Method

The theoretical definition of the WL method of one-dimensional signals is as follows [15,25].

**Step 1.** Calculate the detailed coefficients $d_{X}(j,k)$ of the signals $X=\{X_{i},i=1,2,3,\ldots ,N\}$ by the discrete wavelet transform (DWT), where j and k are the scaling (dilation) index and the shifting (translation) index, respectively. Let ${\left\{X(t)\right\}}_{t\in \mathbb{R}}$ denote the signal $X=\{X_{i},i=1,2,3,\ldots ,N\}$ to be analyzed. Let ψ denote the mother wavelet, characterized by its uniform regularity index and number of vanishing moments $N_{\psi }$, a strictly positive integer defined as $\psi \in C^{N_{\psi }-1}$ and $\forall n=0,\cdots ,N_{\psi -1},\int_{\mathbb{R}}t^{k}\psi (t)dt\equiv 0$ $\int_{\mathbb{R}}t^{N_{\psi }}\psi (t)dt\neq 0$. Let $\{\psi_{j,k}(t)=2^{-j/2}\psi_{0}(2^{-j}t-k),$ $j\in \mathbb{Z},k\in \mathbb{N}{\}}_{(j,k)\in {\mathbb{N}}^{2}}$ denote the collection of dilated and translated templates of ψ that form an orthonormal basis of $L^{2}(\mathbb{R})$. The coefficients of the discrete DWT of X are defined as $c_{j,k}=\langle \psi_{j,k}|X\rangle$. The ($L^{1}$-normalized) discrete wavelet transform coefficients $d_{X}(j,k)=2^{-j/2}\langle \psi_{j,k}|X\rangle$ [26]. Note the use of an $L^{1}$-normalization for the wavelet coefficients that better fits local regularity analysis and yields the correct self-similarity exponent of the wavelet coefficients for self-similar functions [27]. For a detailed introduction to wavelet transforms, readers are referred to, e.g., [28].

**Step 2.** Calculate wavelet leaders $L_{X}(j,k)$. Let us define dyadic intervals as $\lambda_{j,k}=[k2^{j},(k+1)2^{j})$. In addition, let 3λ denote the union of the interval λ with its two adjacent dyadic intervals: $3\lambda_{j,k}=\lambda_{j,k-1}\cup \lambda_{j,k}\cup \lambda_{j,k+1}.$ We define wavelet leaders as

(3) $$L_{X}(j,k)=\underset{\lambda^{\prime }\subset 3\lambda }{\sup}\left|d_{X,\lambda^{\prime }}\right|.$$

This definition means that the wavelet Leader $L_{X}(j,k)$ consists of the largest wavelet coefficient $\left|d_{X}(j^{\prime },k^{\prime })\right|$ computed at all finer scales $2^{j^{\prime }}\leq 2^{j}$ within a narrow time neighborhood, $(k-1)\cdot 2^{j^{\prime }}\leq 2^{j^{\prime }}k^{\prime }<(k+2)\cdot 2^{j}$.

**Step 3.** Calculate the wavelet leaders structure functions $S^{L}(j,q)$. For fixed analysis scales $s=2^{j}$, we can form the time (space) averages of (the q-orders of) the $L_{X}(j,k)$, referred to as the structure functions

(4) $$S^{L}(j,q)=\frac{1}{n_{j}}\sum_{k=1}^{n_{j}}{\left(L_{X}(j,k)\right)}^{q}.$$

where $n_{j}$ denotes the number of $L_{X}(j,k)$ available at scale $2^{j}$.

If the wavelet leaders structure functions $S^{L}(j,q)$ possess power law behaviors with respect to scales in the limit of small scales $s=2^{j}\to 0$

(5) $$S^{L}(j,q)∼s^{\zeta^{L}(q)}.$$

where $\zeta^{L}(q)$ is often referred to as the scaling function. This power law behavior establishes a clear and deep connection between the concepts of scale invariance and multifractal analysis [29].

**Step 4.** Calculate $\zeta^{L}(q)$ with a given q. The scaling function $\zeta^{L}(q)$ is then defined as

(6) $$\zeta^{L}(q)=\underset{s\to 0}{\lim\inf}\frac{\log_{10}(S^{L}(j,q))}{\log_{10}(s)}.$$

It is easy to obtain the singularity strength function α(q) and the multifractal spectrum f(α) via the Legendre transform

(7) $$\alpha (q)=d\zeta^{L}(q)/dq.$$

(8) $$f(\alpha )=\inf_{q}[q\alpha (q)-\zeta^{L}(q)+1].$$

The methods based on the WL with different mother wavelets have different detrending capabilities [30]. In this paper, the wavelet basis function is ‘db3′, and the scaling range is $s=[2^{1},2^{2},2^{3},2^{4},\ldots ,2^{\left\lfloor \log_{2}(N/(2*3+1))\right\rfloor }]$ [26,27].

**Figure 4:**
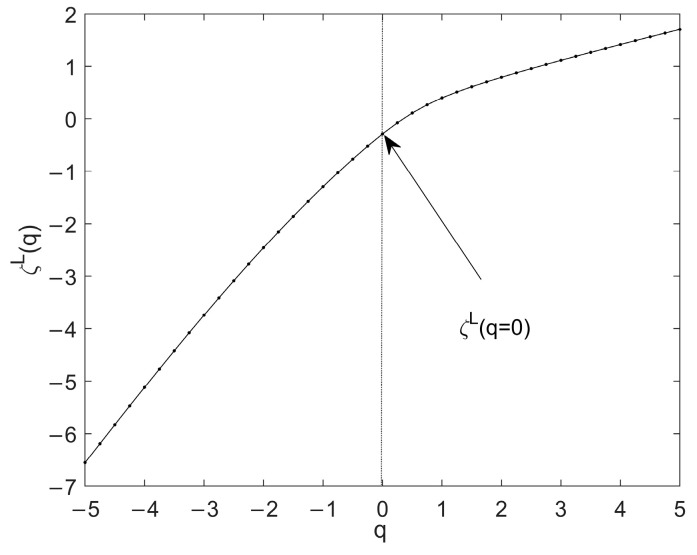
The scaling function $\zeta^{L}(q)$ of the time series based on WL.

A schematic representation of the $q-\zeta^{L}(q)$ curve of one time series is shown in Figure 4, where an obvious inflection point appears near q=0. This means that $\zeta^{L}(q)$ is not a linear function of q, so the time series is multifractal.

**Figure 5:**
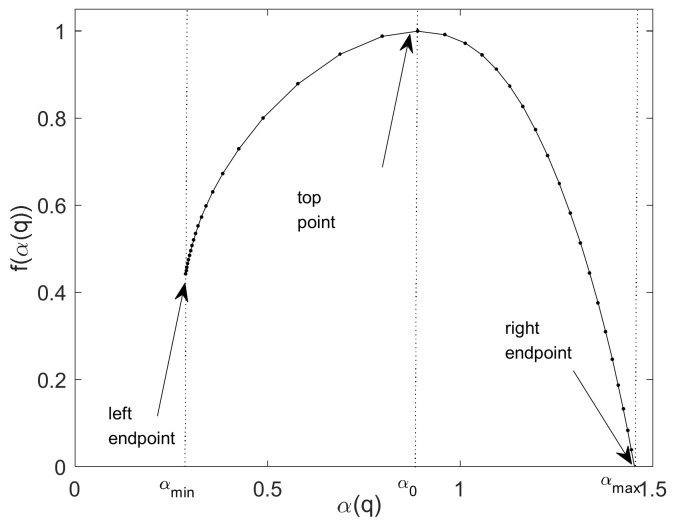
The multifractal spectrum f(α) of the time series based on WL.

Figure 5 shows a schematic representation of the multifractal spectrum f(α) of the same time series based on WL in Figure 4. For the left endpoint of the multifractal spectrum, we use the singularity exponent $\alpha_{\min}$ to denote the corresponding horizontal axis of this point; it reflects the degree of large fluctuation of the signals. $\alpha_{0}$ is the singularity exponent of the top point of the multifractal spectrum and corresponds to the most probable probability subset of the time series. The right endpoint with $\alpha_{\max}$, reflects the degree of small fluctuation of the signals. The spectrum width $\Delta \alpha =\alpha_{\max}-\alpha_{\min}$ reflects the uneven degree of fluctuation of the signals.

For fast speed calculation, we use the matrix calculation method of entropy analysis [31].

(9) $$F_{\alpha (q)}(L_{X}^{q}(j,k))=\frac{\sum_{k=1}^{n_{j}}(L_{X}^{q}(j,k)\log_{2}(L_{X}(j,k)))}{\left(\sum_{k=1}^{n_{j}}L_{X}^{q}(j,k)\right)}=\sum_{k=1}^{n_{j}}[P_{j,k}^{q}\log_{2}(L_{X}(j,k))]∼\log_{2}s^{\alpha (q)}.$$

(10) $$F_{f(\alpha (q))}(L_{X}^{q}(j,k))=\sum_{k=1}^{n_{j}}(P_{j,k}^{q}\cdot \log_{2}P_{j,k}^{q})+\log_{2}(n_{j})∼\log_{2}s^{f(\alpha (q))}.$$

where $P_{j,k}^{q}=\frac{L^{q}(j,k)}{\left(\sum_{k=1}^{n_{j}}L^{q}(j,k)\right)}$.

Similar to the estimation of $\zeta^{L}(q)$ in WL method, α(q) and f(α(q)) are estimated as the linear regression slopes of the q-order entropies $F_{\alpha (q)}(L_{X}^{q}(j,k))$ and $F_{f(\alpha (q))}(L_{X}^{q}(j,k))$, respectively, and scale $s=2^{j}$ in log-log coordinates.

#### 2.2.5. Extreme Learning Machine

**Figure 6:**
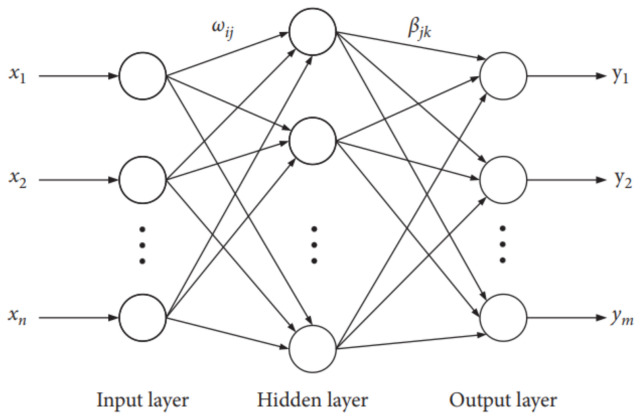
Structure of basic ELM.

The Extreme Learning Machine (ELM) is a single implicit layer feedforward neural network [32,33]. By setting the number of neurons in the implicit layer, the connection weights β between the implicit layer and the output layer are not adjusted iteratively but are determined once by solving a system of equations. From the perspective of learning efficiency, the extreme learning machine has the advantages of few training parameters, fast learning speed, and strong generalization ability. It consists of three parts: the input layer, the implicit layer, and the output layer, as shown in Figure 6. In this paper, we use the ELM classifier to diagnose CHF.

#### 2.2.6. K-Fold Cross-Validation

In recent years, K-fold cross-validation has been commonly used in applied machine learning to compare and select models for a given predictive modeling problem. This is easy to understand and implement, resulting in skill estimates that typically have lower bias than other methods. In our experiments, the data were divided into K (K = 5) equal-sized parts, with one of the five parts selected for testing and the rest of the data for training. The results recorded in all five iterations are averaged and considered as the overall performance of our proposed system. For all class-oriented experiments, we used this cross-validation method.

#### 2.2.7. Evaluation Criteria

Accuracy alone is not sufficient in the classification of data, as it is calculated by the ratio of accurately estimated data to the total data set. Sensitivity measures how often a test works properly, while specificity is a measure of a test’s ability to produce negative results for an untested disease. Therefore, these parameters should be assessed together. On the other hand, F1 score is the summed average of precision and recall rather than the arithmetic means to avoid ignoring extremes. Therefore, F1 score must also be included in the assessment metric. The equations for the assessment indicator are as follows:

•Accuracy:

(11) $$ACC=\frac{TP+TN}{TP+TN+FP+FN}\times 100\%$$

•Precision:

(12) $$PPV=\frac{TP}{TP+FP}\times 100\%$$

•Sensitivity:

(13) $$SEN=\frac{TP}{TP+FN}\times 100\%$$

•Specificity:

(14) $$SPE=\frac{TN}{FP+TN}\times 100\%$$

•F1 score:

(15) $$F1=\frac{2TP}{2TP+FN+FP}\times 100\%$$

where *TP* indicates correct identification in the absence of disease, *TN* indicates correct detection of disease, *FP* indicates incorrect detection when the disease is present, and the detector is not detected, and *FN* indicates that disease is not present, but the detector detects disease.

## 3. Results

### 3.1. Optimization of Parameter Settings

When we use the MSE method, we first need to determine the parameter settings (s, m, r). Usually, the length of the data should be at least $10^{m}–20^{m}$ [34]. When MSE is used to analyze ECG signals, the length of the data $N=t\cdot f_{s}$, $f_{s}$ is the sampling frequency of the ECG signals, so we need to determine the segmentation time t of the ECG, The parameters r should be 10–20% [24] of the standard deviation, and we now use MSEN1 as an example to illustrate the effect of the parameter settings.

#### 3.1.1. Embedded Dimensions

As with approximate entropy and sample entropy, the embedding dimension m=2 is generally taken. The larger m is, the more detailed information can be obtained when dynamically reconstructing the joint probabilities, but the larger m is, the longer the length of data required, and the computation time will be longer. Therefore, we use the embedding dimension m=2.

#### 3.1.2. Segmentation Time

In the analysis and processing of real signals, the length of the signal segmentation is an important factor affecting real-time analysis. If its features can be obtained with shorter signals, it will be important for the diagnosis of diseases. The sampling frequency of the NSR dataset is 128 Hz, and it is known that the data length N should be greater than 256, $t=N/f_{s}=256/128=2$ s, so the time t=[4,8,16,32,64,128] s is chosen for testing. After testing, it was found that there are a large number of INF points for calculating the multiscale entropy when segmentation time t is less than 32 s, which is because the multiscale entropy algorithm gives an inaccurate estimate of entropy, even leading to short time sequences with undefined entropy values. Therefore, the segmentation time t=[32,64,128] s were chosen.

**Figure 7:**
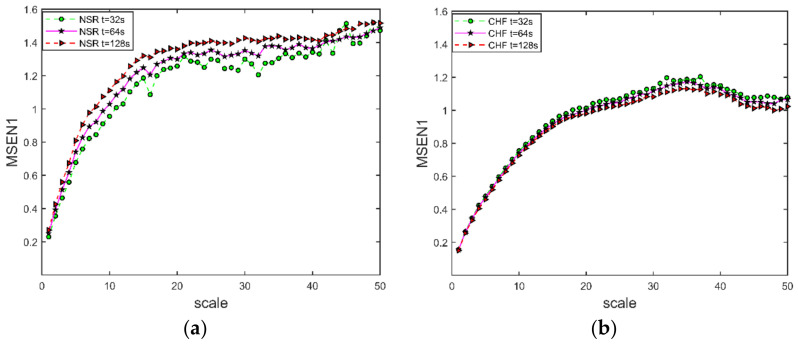
MSEN1 curve at different segmentation time. (**a**) NSR ECG signal; (**b**) CHF ECG signal. The embedding dimension m=2, scale s=1:1:50, and similarity tolerance r=0.2σ (σ is the standard deviation of the time series after coarse granulation).

The curves of MSEN1 for the ECG signals of NSR and CHF at t=[32,64,128] s are shown in Figure 7, where s=1:1:50, m=2, and r=0.20σ. As can be seen from Figure 7a,b, the differences between the three curves for different segmentation times are very small. Therefore, the length of the segmentation time has little effect on the multiscale entropy of the ECG signals. The multiscale entropy of the NSR data is unstable at t=32 s and more stable at t=64 s and t=128 s. However, the larger the segmentation time, the more sampling points, and the longer the calculation time; therefore, the segmentation time is chosen to be t=64 s.

The ECG signals for both data were chosen to be segmented at time t=64 s. The datasets used in this study (Set A, Set B) are shown in Table 1. Both sets A and B contain full ECG data, with Set A being the unbalanced dataset and Set B being the balanced dataset.

#### 3.1.3. Scale Factor

**Figure 8:**
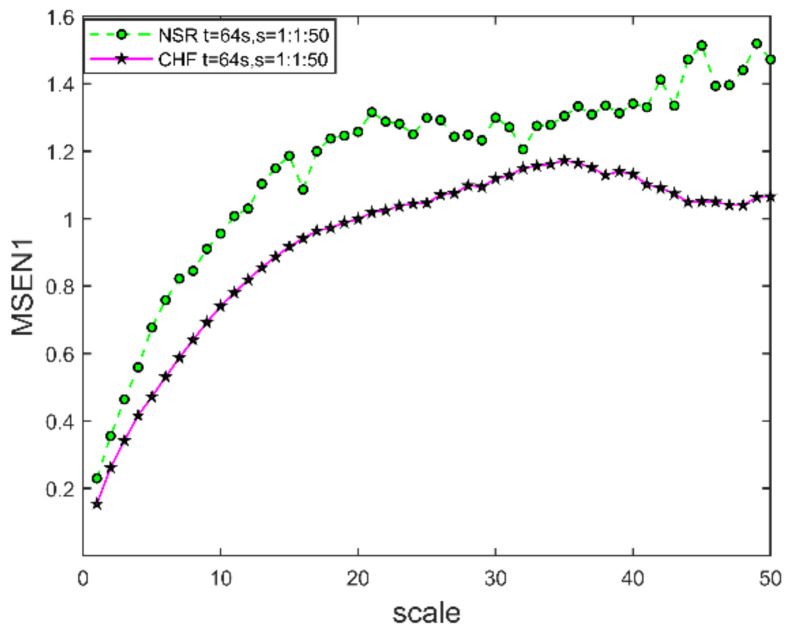
MSEN1 curves for two ECG signals at different scales. The embedding dimension m=2, segmentation time t=64 s, and similarity tolerance r=0.2σ.

We plotted the MSEN1 curve for the ECG signals at scale s=1:1:50 as shown in Figure 8, where t=64 s and m=2. The entropy values of the coarse-grained time series from healthy subjects are significantly higher than those of the CHF. Therefore, coarse-grained time series from healthy subjects at large time scales may be more complex. This finding is consistent with 1/f noise [4] (1/f noise is generated as follows: we start with uniformly distributed white noise, we compute the fast Fourier transform (FFT), and after imposing an 1/f distribution on the power spectrum, we compute the inverse FFT.) containing complex structures on multiple time scales. We use entropy methods to quantify the dynamics of each coarse-grained time series. We found an overall increasing trend in the magnitude of entropy values in the scale 1 to 20 range and stabilization to relatively constant values in the scale 20 to 50 range. However, the entropy values of coarse-grained time series from NSR were significantly higher than those of CHF. This suggests that the normal ECG signal data is more complex and the ECG signal from congestive heart failure is more regular.

When the selected sampling points are fixed, the larger the scale s, the less time is required for the calculation of MSEN1. However, with too large a scale value, the final data will become shorter, and the number of vector groups obtained in the coarse-grained process will be smaller. Therefore, the scale should be relatively small, and MSEN1 clearly varies linearly as the scale s. Therefore, we choose scale s=10:1:20.

#### 3.1.4. Similarity Tolerance

**Figure 9:**
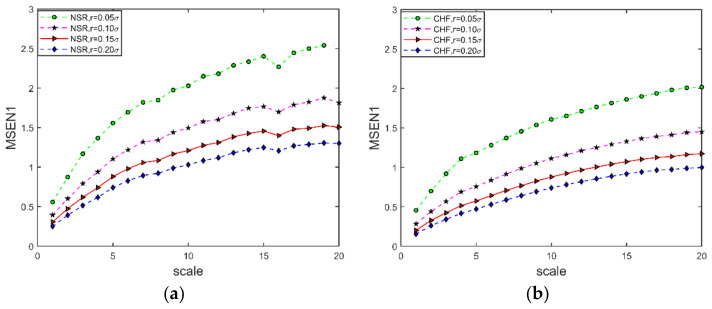
MSEN1 curve at different similarity tolerance. (**a**) NSR ECG signal; (**b**) CHF ECG signal. The embedding dimension m=2, segmentation time t=64 s, and scale s=1:1:20.

The similarity tolerance r represents the width of the fuzzy function boundary. r is too large, and much statistical information is lost; r is too small, and the estimated statistical properties are unsatisfactory, increasing sensitivity to the noise of the results. Figure 9 shows the MSEN1 curves for the ECG signals of NSR and CHF for different r=[0.05σ,0.10σ,0.15σ,0.20σ] at the t=64 s and s=1:1:50. For Figure 9a, the green dashed line with the green circle indicates that the mean of the MSEN1 of the signals with r=0.05σ (σ denotes the standard deviation of the time series after coarse granulation) has an omission because r is too small and the statistical properties are not ideal. From Figure 9a,b, it can be seen that the larger the similarity tolerance r is, the larger the multiscale entropy value is, and r=0.15σ is chosen to maintain the smoothness of the data.

#### 3.1.5. Multifractal Spectrum Features

The mother wavelets ${\left\{\psi^{\left(i\right)}(x)\right\}}_{i=1,\cdots ,2^{d}-1}$ are further required to possess additional regularity and localization properties. They are assumed to belong to $C^{r_{\psi }}({\mathbb{R}}^{d})$ with $r_{\psi }$ as large as possible. When $r_{\psi }\geq 1$, all their partial derivatives of order at most $r_{\psi }$ have fast decay. For a one-dimensional signal, we have d=1. In addition, the number of vanishing moments $N_{\psi }$ is defined as a positive integer such that for any polynomial P of degree strictly smaller than $N_{\psi }$, $\int_{\mathbb{R}}P(x)\psi^{\left(i\right)}(x)dx=0$. Both the regularity and the vanishing moment assumptions are required in order to obtain accurate wavelet characterizations of pointwise regularity: Let $h_{\max}$ denote the largest smoothness order found in X, then a sufficient condition for choosing the mother wavelet reads:$h_{\max}<\min(r_{\psi },N_{\psi })$.

The computation of the uniform Hölder exponent $h_{\min}$ [35], using the following wavelet characterization: $h_{\min}=\underset{j\to -\infty }{\lim\inf}\frac{\log_{2}(\underset{k}{\sup}\left|d_{X}(j,k)\right|)}{\log_{2}(2^{j})}$. Indeed, if $h_{\min}>0$, then X is a continuous function, whereas if $h_{\min}<0$, then $X\notin L_{loc}^{\infty }$ see References [36,37]. For numerous real-world applications, the restriction $h_{\min}>0$ constitutes a severe limitation, c.f. Reference [37].

From a practical point of view, mother wavelets satisfying $r_{\psi }>h_{\min}$ are required for an accurate estimation of $h_{\min}$. Similarly, $h_{\max}=\underset{j\to -\infty }{\lim\inf}\frac{\log_{2}(\underset{k}{\min}\left|d_{X}(j,k)\right|)}{\log_{2}(2^{j})}$.

In general, one does not have information concerning the a priori regularity of the data. Therefore, one does not know how smooth the analyzing wavelets should be. In practice, a rule of thumb consists of using smoother and smoother wavelets until the outcome no longer depends on the wavelet used, which is interpreted as meaning that sufficient regularity has been reached. This can afterward be confirmed using multifractal analysis tools (see Reference [36]), which allow us to determine the maximum regularity exponent present in the data. Further, with orthonormal wavelet bases (such as the so-called “Daubechies” compactly supported wavelets), widely used in applications), one necessarily has $N_{\psi }\geq r_{\psi }$. A sufficient (and conservative) condition for accurate wavelet characterizations of pointwise regularity simplifiers to $h_{\max}<r_{\psi }$.

**Figure 10:**
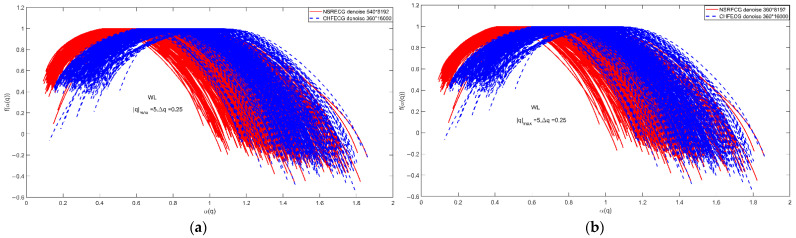
WL-based multifractal spectrum. (**a**) Set A; (**b**) Set b. The ‘db3’ wavelet basis functions ${\left|q\right|}_{\max}=5$, Δq=0.25.

We adhere to the convention that the finest available dyadic scale is labeled by $j_{1}=1$. That means $s=2^{1}$, 1-layer wavelet decomposition. A Daubechies’ wavelet with $N_{\psi }=3$ vanishing moments is used, then $j_{2}=10=\left\lfloor \log_{2}(8192/(2*3+1))\right\rfloor$, this means $s=2^{10}$, 10-layer wavelet decomposition. Because $\alpha_{\min}>0$, $\alpha_{\max}<3$ in Figure 10a,b, for $h_{\min}>0$ and $h_{\max}<3$, so ‘db3′ is suitable. Figure 10 shows the simulation results for data sets A and B. In this paper, the WL-based multifractal analysis method is used, where the ‘db3’ wavelet basis functions are ${\left|q\right|}_{\max}=5$, Δq=0.25, and the entropy analysis is carried out by matrix calculation methods, and the scale range can be $s=[2^{1},2^{2},2^{3},2^{4},2^{5},2^{6},2^{7},2^{8},2^{9},2^{10}]$.

Figure 10a shows the WL-based multifractal spectrum for set A with 540 groups of NSR ECG signals of 8192 points and 360 groups of CHF ECG signals of 16,000 points, where the ‘db3’ wavelets are ${\left|q\right|}_{\max}=5$ and Δq=0.25. For ‘db3’, the number 3 refers to the number of vanishing moments. The NSR and CHF signals are sampled by different sample frequencies, so we choose the signal of 64 s (8192/128 = 64, 16,000/250 = 64) for NSR and CHF ECG signals, which results in the two different lengths of the signals 8192 and 16,000. When we do the simulations, we choose the same parameters for them, so the scale range is determined by the length 8192. The length of the wavelet filter is 2×3=6 of ‘db3’. Thus, when we choose the parameter $s=[2^{j_{1}},\cdots ,2^{j_{2}}]$ for the range of scales, we fix the maximum scale $2^{j_{2}}$ by choosing the largest integer $j_{2}$ that is not greater than $\left\lfloor \log_{2}(N/(2\times 3+1))\right\rfloor$, i.e., $\left\lfloor \log_{2}(8192/(2\times 3+1))\right\rfloor$ ≈13−3=10. In the simulations, we use $j_{2}=10$ and $j_{1}=1$. Figure 10b shows the WL-based multifractal spectrum for set B, where the ‘db3’ wavelet is ${\left|q\right|}_{\max}=5$, Δq=0.25 and there are differences between the two ECG signals of the same sample numbers 360 groups, respectively.

In Figure 3 after wavelet denoising, the small fluctuations are removed, and the slightly larger fluctuations are retained, as reflected by the singularity index $\alpha_{\min}=\alpha (q=5)$ at the left endpoint of each multifractal spectrum. These large fluctuations in the ECG signals of a normal person are similar to each other, but the fluctuations in the ECG signals of an unhealthy person are not uniform and regular, so the slightly larger fluctuations result in slightly smaller singularity exponents of the left end point of the multifractal spectrum. When q>>1, the large fluctuations will dominate the statistics and result in a smaller singularity index. The singularity index $\alpha_{0}$ corresponding to the top of the multifractal spectrum corresponds to the most probable or most likely subset (i.e., the subset with the largest number of line segments or elements, and this subset has a fractal dimension of 1, the ordinal number of points at the top of the multifractal singularity spectrum). The calculated multifractal profiles for normal and unhealthy individuals have some deviation in the value of the singularity index corresponding to the top point of the multifractal profile. When we extract the multifractal spectrum using the WL method, we can use the two parameters $\alpha_{\min}$ and $\alpha_{0}$ to do the classification of the ECG signals of normal and unhealthy individuals.

#### 3.1.6. Number of ELM Hidden Layer Nodes

**Figure 11:**
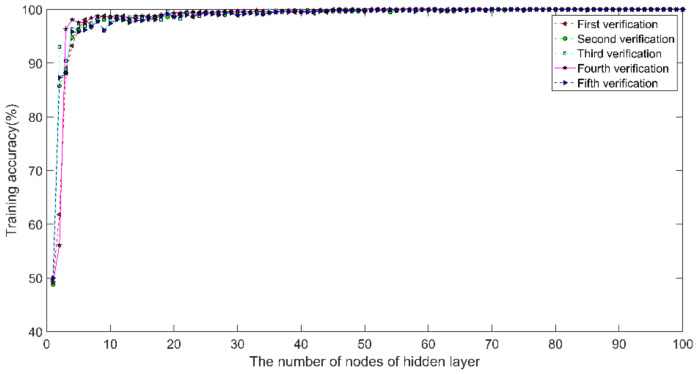
Classification accuracy for 5-fold cross-validation.

In this study, ELM was selected as the classifier. To maintain good generalization performance, it was particularly important to determine l, which denotes the appropriate number of nodes of the hidden layer. The optimal number of nodes of the hidden layer was determined using five-fold cross-validation. The balanced dataset Set B was selected, and the 360 ECG segments were divided into five parts, of which four were used for training the model and one for testing the model. MSEN1, MSEN2, and the singularity indices $\alpha_{0}$ and $\alpha_{\min}$ of the multifractal spectra of Group B were calculated according to the multiscale entropy algorithm for the first-order moment and second-order moment and WL. The number of nodes of the hidden layer was 1–100. The classification accuracy of the training set was obtained from five-fold cross-validation, as shown in Figure 11.

It can be seen from Figure 11 that the classification accuracy of the training and test sets gradually increases as the number of nodes of the hidden layer increases. The accuracy of the five-times cross-validation gradually increases to 100% when l>20. The classification accuracy of the training set with node numbers from 20 to 70 is shown in Table 2. It can be observed from Table 2 that the best results are obtained for the 5-fold training set when l≥60. Considering the running time, l=60 is chosen as the optimal number of hidden layer nodes.

### 3.2. Training and Test of the CHF Classifier

#### 3.2.1. Results of Classification

The number of ECG segments in sets A and B, the parameter settings of the generalized multiscale entropy algorithm, the WL-based multifractal spectrum parameters, and the number of ELM hidden layer nodes are determined in Section 3.1. Finally, $\left[MSEN1,MSEN2,\alpha_{0},\alpha_{\min}\right]$ feature vectors are fed into the classifier ELM with 60 nodes of the hidden layer for classification. A five-fold cross-validation strategy is used to evaluate diagnostic algorithms using the accuracy, precision, sensitivity, specificity, and F1-score parameters obtained from the confusion matrix parameters.

The ECG segments of sets A and B were divided equally into five segments. For each iteration, four of five ECG segments were selected for training, and the rest were used for testing, which was repeated five times. Finally, the average of the five iterations was taken. Table 3 and Table 4 show the overall average performance of our proposed classification method for classifying normal and CHF categories for both sets A and B.

In Table 3, the parameters of the confusion matrix are shown for the unbalanced data, Set A. Our algorithm achieved significant results for CHF detection, with an accuracy of 99.56%, precision of 99.44%, sensitivity of 99.81%, specificity of 99.17%, and an F1 score of 99.62%. SEN value is 99.81%, which means 99.81% of the normal ECG segments were correctly classified as normal. SPEC is 99.17%, which means 99.17% of the CHF signals were correctly classified as CHF category, and only 0.19% and 0.83% of the ECG signals were incorrectly classified as CHF and normal. In Table 4, for Set B, the overall accuracy of 99.72%, precision of 99.46%, sensitivity of 100%, specificity of 99.44%, and an F1 score of 99.73%. Only 0.56% of ECG signals are incorrectly classified as CHF. It can also be seen that the accuracy of the CHF detection rate for the unbalanced data Set A in Table 3 is less than that of the CHF detection rate for the balanced data Set B in Table 4, with a variability of less than 0.3%.

#### 3.2.2. Results of Adding Data Segments

In order to evaluate the effectiveness of the algorithm and model proposed in this paper for CHF detection and to validate the CHF detection rate of the unbalanced data set, the number of ECG signal segments was increased. 1800 ECG segments were taken for NSR, and 1500 ECG segments were taken for CHF, set as Set C.7200 ECG segments were taken for NSR, and 6000 ECG segments were taken for CHF, set as Set D. The overall confusion matrix of CHF detection with the same parameters selected for the same algorithm is shown in Table 5. For Set C, the overall accuracy of 99.24%, precision of 99.22%, sensitivity of 99.39%, specificity of 99.07%, and an F1 score of 99.30%. Set D attained an accuracy of 99.41%, precision of 99.57%, sensitivity of 99.35%, specificity of 99.48%, and an F1 score of 99.46%. The differences in the five diagnostic assessment indicators were smaller in Sets C and D compared with Set A, with the largest difference in specificity being 0.65%.

Table 6 shows the time required to run each dataset. The more ECG signal segments there are, the more time it takes to diagnose CHF. Therefore, the increase in data segments did not significantly reduce the accuracy of diagnosing CHF, but the training time increased, and the training time was shorter in Set A. Our proposed algorithm can therefore select a small number of ECG time series to diagnose CHF in the presence of a large amount of data and significantly improves the accuracy of diagnosing unbalanced data sets.

#### 3.2.3. Comparison Results of Different Algorithms

Data Set A was selected, and the same five-fold cross-validation strategy was used; four of five ECG segments were selected for training, and the rest were used for testing. The generalized multiscale entropy extracted features and WL-extracted features are fed into ELM classification separately, and then the diagnostic results by using the classifiers Support Vector Machine (SVM) and K-Nearest Neighbor (KNN) are compared with the results of the proposed diagnose method in this paper. For the multi-classification of ECG signals by using SVM and Support Regression Machine (SVR), we used the kernel function as Radial Basis Function, and the gamma parameter coefficient was 0.05. For the KNN algorithm, the K value was chosen as the square root of the number of samples in the training set, which was 30.

**Figure 12:**
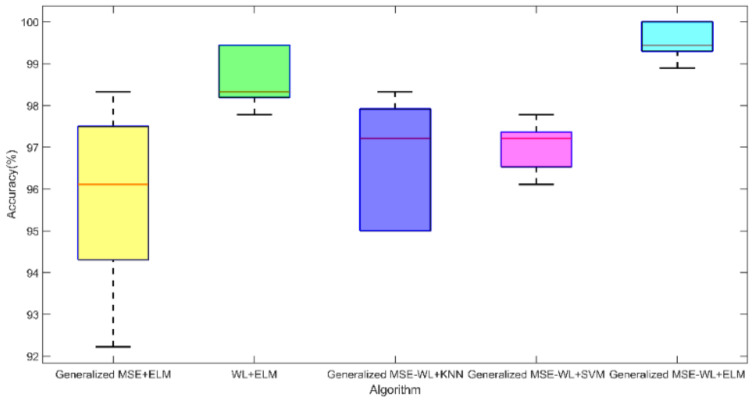
Block diagram of accuracy of different algorithms.

The results of 5-fold cross-validation are shown in Table 7, and the associated box plot is shown in Figure 12 to display the influences of the different algorithms on the accuracy of diagnostic results. One can conclude that each classifier has high accuracy (>95%) because of the distinguishable feature extracted in the previous stage of work. The highest overall accuracy of 99.56% correct detection of CHF is obtained in the results of the generalized MSE-WL-based detection method with ELM classification. Therefore, based on the overall classification correctness, it is concluded that the generalized MSE-WL feature extraction method can be applied to CHF automatic diagnosis. The extracted features have a good performance on multiple classifiers.

## 4. Discussion

According to the results obtained in Table 7, the method is better for classifying ECG signals of NSR and CHF compared with the algorithms based on the physiological MIT-BIH database. Table 8 shows a comparison of the results obtained with the literature of recent years. In the literature, CHF and NSR conditions are classified using classical machine learning for the classification algorithm. There is literature to detect CHF ECG signals with accuracy greater than 99%, but most select a large number of ECG segments, and some detect R peaks. In contrast, we proposed a diagnosis method of CHF; it does not need to detect R-peaks on the ECG signals and extract a small number of ECG segments to obtain a CHF detection rate greater than 99% and to improve the accuracy of the unbalanced dataset. In this study, the time series of ECG signals used was short. Although the proposed feature extraction method did not achieve 100% accuracy in detecting CHF, it was the first implementation to combine generalized multiscale entropy and WL algorithms to classify ECG signals into normal and CHF categories using the ELM classifier, with 99.17% (Set A) and 99.44% (Set B) specificity in detecting CHF. With no reduction in CHF detection performance, the method does not require R-wave extraction. As a complexity analysis method, the generalized multiscale entropy method does not strongly depend on the data length when dealing with different complex signals. Therefore, the method requires fewer ECG segments to detect CHF and improves the timeliness of heart failure diagnosis.

In this work, we selected ECG signals with a duration of 64 s for automatic diagnosis of CHF using a feature binding approach. The programming implementations of the generalized multiscale entropy and WL algorithms are based on entropy theory. The former evaluates the complexity of a time series by quantifying its entropy over a range of temporal scales, and the coarse-grained procedure of different scale values results in a longer run time. However, the programming procedure of the linear fit of the power-law relationship of different scale values in the WL algorithm, which is realized with the help of matrices, runs fast. Hence, compared with other algorithms in the literature, the novelty of this work is to combine the generalized multiscale algorithm with the WL algorithm to obtain good feature vectors to ensure comparable good performance of the diagnosis of CHF, which will help cardiologists in the diagnosis and treatment of CHF.

The advantages of our proposed classification method are: (1) A feature classification method of CHF and NSR signals based on the generalized MSE-WL and ELM is proposed. (2) No R-peak detection is required. (3) Requires a small number of ECG segments. (4) Improves the detection rate of unbalanced datasets. The limitations of the proposed classification method are: (1) Requires ECG segmentation time greater than 32 s. (2) No tests for other cardiac diseases were performed.

## 5. Conclusions

CHF is a complex clinical condition in which the ability of the heart to fill and pump blood is impaired due to functional or structural disease. Early detection of CHF is of high importance to avoid death. In this paper, we propose a CHF feature classification method based on generalized MSE-WL and ELM, which does not require R-peak detection and uses ECG signals to diagnose CHF automatically. The ECG signals obtained from Physionet are used to determine the parameter settings for the first moment of MSE by simulations of different data segmentation, scaling ranges, and similarity tolerance. It provides guidance on feature selection and parameter settings in practical applications. The extracted feature factors are fed into the ELM for training and testing, and confusion matrices and accuracy values are given to evaluate the results obtained. Using our proposed method, we obtained the balanced data set (B) had an accuracy of 99.72%, and the unbalanced data set (A) attained an accuracy of 99.56%. The results of CHF classification suggest that a CHF detection method based on generalized MSE-WL can help doctors better diagnose CHF. The method requires fewer ECG segments to accurately distinguish between normal and CHF patients and can considerably reduce the workloads of doctors. It provides clinicians with a valuable reference for diagnosing CHF.

In the future, we will investigate a new method to automatically detect cardiac disease by imaging the ECG signal without removing noise. The ECG signal will be characterized using two-dimensional entropy theories and two-dimensional multifractal methods [43]. Then a classification method will be used to classify the ECG signal and diagnose cardiac disease.

## Figures and Tables

**Table 1:** Total number of ECG segments used in the data set.

	Number of 64 s ECG Segments	
Type (Database)	Unbalanced	Balanced
	A	B
Normal (NSR)	540	360
CHF (BIDMC)	360	360

**Table 2:** Training accuracy of ELM with the number of nodes of the hidden layer.

			Number of ELM Hidden Layer Nodes (%)								
K-Fold	20	25	30	35	40	45	50	55	60	65	70
1	98.61	99.48	98.96	99.65	99.65	100	99.65	99.83	100	100	100
2	98.78	99.48	99.48	99.31	99.48	99.65	99.83	99.83	100	100	100
3	98.96	99.48	99.65	99.83	99.83	99.65	99.65	99.83	100	99.83	99.65
4	99.31	99.48	99.65	99.48	99.48	99.83	100	100	100	100	100
5	98.61	99.13	98.96	99.48	99.65	99.83	99.65	99.83	99.83	100	100

**Table 3:** Confusion matrix for unbalanced data set-NSR/CHF (Set A).

		Predicted						
		Normal	CHF	ACC (%)	PPV (%)	SEN (%)	SPEC (%)	F1 (%)
Original	Normal	539	1	99.56	99.44	99.81	99.17	99.62
	CHF	3	357					

**Table 4:** Confusion matrix for balanced data set-NSR/CHF (Set B).

		Predicted						
		Normal	CHF	ACC (%)	PPV (%)	SEN (%)	SPEC (%)	F1 (%)
Original	Normal	360	0	99.72	99.46	100	99.44	99.73
	CHF	2	358					

**Table 5:** Classification results of CHF detection categories with different ECG fragments.

Dataset	TP	TN	FP	FN	ACC (%)	PPV (%)	SEN (%)	SPEC (%)	F1 (%)
A	539	357	3	1	99.56	99.44	99.81	99.17	99.62
C	1789	1486	14	11	99.24	99.22	99.39	99.07	99.30
D	7153	5969	31	47	99.41	99.57	99.35	99.48	99.46

**Table 6:** Running time to complete a dataset.

Dataset	Running Time (s)
A	731.45
C	3107.80
D	11,825.24

**Table 7:** Accuracy of 5-fold classification for different algorithms.

Algorithm	Fold1 (%)	Fold2 (%)	Fold3 (%)	Fold4 (%)	Fold5 (%)	OA (%)
Generalized MSE + ELM	92.22	98.33	97.22	96.11	95.00	95.78
WL + ELM	98.33	99.44	98.33	97.78	99.44	98.67
Generalized MSE-WL + KNN	95.00	98.33	95.00	97.78	97.22	96.67
Generalized MSE-WL + SVM	97.22	97.78	97.22	96.67	96.11	97.00
Generalized MSE-WL + ELM (Set A)	99.44	100	100	98.89	99.44	99.56

**Table 8:** Literature Comparison.

Reference	Year	Number of ECG Data	Method	Performance
Daqroup and Dobaie [38]	2016	CHF: 140 Normal: 152	▪Wavelet Packet Transform ▪Feature Extraction	Acc—92.60%
Sundarshan et al. [39]	2017	CHF: 25,328 Normal: 59,624 CHF: 25,328 Normal: 25,328	▪Denoising and baseline removal ▪Dual tree complex wavelet transform ▪KNN classifier (2-s ECG segment)	Acc—98.42% Sen—97.04% Spec—99.01% Acc—97.94% Sen—98.19% Spec—97.69%
Acharya et al. [17]	2018	CHF: 30,000 Normal: 70,308 CHF: 30,000 Normal: 30,000	▪KNN classifier ▪11-layer deep CNN (2-s ECG segment)	Acc—95.98% Sen—96.52% Spec—95.75% Acc—94.40% Sen—94.68% Spec—94.12%
Jahmunah et al. [16]	2019	CHF: 30,000 Normal: 70,308 CHF: 30,000 Normal: 30,000	▪Fuzzy entropy ▪Rényi entropy ▪Higuchi Fractal Dimension ▪Kraskov entropy, energy ▪Frequency localized filter banks ▪Quadratic support vector machine (QSVM) ▪10-fold cross validation (2-s ECG segment)	Acc: > 99.66% Sen: > 99.82% Spec: > 99.28%
Yue Zhang and Ming Xia [40]	2020	CHF: 53,857 Normal: 58,675	▪Detected R peaks ▪RNN	Acc = 99.17% Sen = 99.40% Spec = 98.96%
Taotao Liu et al. [41]	2022	CHF: 36,000 Normal: 30,000	▪Feature Extraction ▪ECVT-Net ▪CNN	Acc: 98.88% Pre: 98.84% Sen: 98.94%
Zeming Liu et al. [42]	2022	1 min length of RR segment	▪Multi-feature—fApEn_IBS + IBS + LF/HF ▪Random Forces	Acc = 99.0% Sen = 97.8% Spec = 100.0%
Proposed Method	2022	CHF: 540 Normal: 360 CHF: 360 Normal: 360	▪The generalized multiscale entropy (MSE) ▪Wavelet leaders (WL) ▪ELM classifier (64-s ECG segment)	Acc—99.56% Sen—99.81% Spec—99.17% Acc—99.72% Sen—100% Spec—99.44%

## Data Availability

All data used in the experiments can be downloaded from the following links: http://www.physionet.org/physiobank/database/#ecg (accessed on 15 June 2022).
